# Treatment Landscape for Older Men With Metastatic Hormone‐Sensitive Prostate Cancer in the United States

**DOI:** 10.1002/cam4.71176

**Published:** 2025-09-03

**Authors:** Bo Zhou, Amit D. Raval, Yifan Zhang, Matthew J. Korn, Nethra Sambamoorthi, Rafia Rasu, Natasha Littleton, Niculae Constantinovici, Usha Sambamoorthi

**Affiliations:** ^1^ University of North Texas Health Science Center Fort Worth Texas USA; ^2^ Bayer HealthCare Pharmaceuticals Whippany New Jersey USA; ^3^ Bayer AG Dexter Michigan USA; ^4^ Bayer Ltd Dublin Ireland; ^5^ Bayer Consumer Care AG Basel Switzerland

**Keywords:** androgen receptor pathway inhibitors (ARPI), metastatic hormone‐sensitive prostate cancer (mHSPC), real‐world evidence (RWE), SEER Medicare, treatment intensification

## Abstract

**Objective:**

This study evaluated treatment patterns and factors associated with androgen deprivation therapy (ADT) intensification with androgen receptor pathway inhibitors (ARPI) and/or docetaxel among older men with mHSPC in the United States.

**Methods:**

The study utilized a retrospective cohort of 6850 older men (age ≥ 67 years) diagnosed with mHSPC between July 2016 and December 2019 from the Surveillance, Epidemiology, and End Results Medicare‐linked database. Men must maintain continuous enrollment in Medicare fee‐for‐service Parts A/B/D for ≥ 12 months before mHSPC diagnosis and ≥ 6 months after diagnosis. Initial treatment was classified as ADT alone, ADT + ARPI, or ADT + docetaxel. Factors associated with initial treatment were examined using multivariable multinomial logistic regression.

**Results:**

The study cohort (mean age = 76.6 years, SD = 7.0) was mostly non‐Hispanic white (77.7%), followed by non‐Hispanic Black (8.4%) and Hispanic (6.5%). 30.4% received no systemic drug therapy within 6 months of mHSPC diagnosis, 47.1% received ADT alone, 14.8% received ADT + ARPI, and 5.9% received ADT + docetaxel. Among men treated with ADT, there was an increase in ADT + ARPI treatment from 19.0% to 30.0% and a concomitant decline in ADT monotherapy from 72.1% to 62.6% between 2017 and 2019, while ADT + docetaxel treatment marginally decreased from 8.8% to 7.3%. In multinomial logistic regression, men with *de novo* mHSPC were more likely to receive ADT + docetaxel (aOR = 2.73, 95% CI = [2.07, 3.60]) or ADT + ARPI (aOR = 1.56, 95% CI = [1.32, 1.84]); whereas men with higher frailty index were less likely to receive ADT + docetaxel (aOR = 0.93, 95% CI = [0.91, 0.95]) or ADT + ARPI (aOR = 0.97, 95% CI = [0.96, 0.98]). Specifically, ADT + ARPI was less likely to be utilized among the lower socio‐economic status groups.

**Conclusions:**

Three in 10 older men with mHSPC received no systemic treatment. Although there was a gradual uptake of ARPIs, monotherapy with ADT was still highly prevalent, suggesting the integration of intensified treatment is still suboptimal. Targeted interventions are necessary to enhance guideline adherence among older and frail men with mHSPC.

## 1 Introduction

Prostate cancer (PC) is the second leading cause of cancer‐related death among men in the United States (US). According to recent national estimates, 288,300 new cases of PC were estimated to be diagnosed in 2023, and 3.5 million cancer survivors had a history of PC in 2020 [1, 2]. Nearly 8% of new cases were diagnosed with metastases within the US [1]. It was estimated that 120,368 men with PC had metastases as of 2018. Nearly 55% of metastatic diseases had de novo metastases/synchronous (i.e., at diagnosis of PC) metastatic hormone‐sensitive PC (mHSPC), while the remaining 45% progressed from non‐metastatic to metastases prior to developing castration resistance [3].

The incidence rates of metastatic PC increased significantly between 2010 and 2018 among men of all age groups [4]. For example, among men aged 75 years or older, the incidence rate of metastatic PC increased from an annual percentage of 6.5% in 2011 to 7.8% in 2018. Among men aged 55–69years, there was a 92% increase in metastatic PC [5]. Hormone‐sensitive PC (HSPC) is the predominant etiology when patients present with metastatic disease at diagnosis [6].

Survival of men with mHSPC has dramatically improved over recent years due to the evolving treatment landscape [7]. Before 2014, the mainstay of treatment for mHSPC was androgen deprivation therapy (ADT) through medications or surgical procedures. Since 2015, multiple clinical trials have shown that doublet or triplet therapy for mHSPC is associated with longer survival. For example, CHAARTED and STAMPEDE trials demonstrated the benefits of combining docetaxel with ADT in progression‐free survival (PFS) and overall survival (OS) [8, 9]. Several trials showed that adding androgen receptor pathway inhibitors (ARPIs), a class of drug that inhibits tumor growth by blocking androgen receptor pathways, to ADT can also improve OS. The doublet therapy included ADT + abiraterone (STAMPEDE and LATITUDE), ADT + apalutamide (TITAN) and ADT + enzalutamide (ENZAMET and ARCHES) [10, 11, 12]. Other randomized controlled trials (RCTs) have shown that triplet therapy (ARPI + ADT+ Docetaxel) compared to doublet therapy (ADT + Docetaxel) further improved OS among men with mHSPC [13, 14].

Given the benefits of intensification, ADT monotherapy is often considered insufficient [15]. Current clinical guidelines recommend doublet or triplet therapies for men with mHSPC as the standard of care [16]. However, findings from studies using real‐world data indicate that many men with mHSPC still receive ADT monotherapy. For example, Ryan et al. examined the treatment patterns in 6517 men with mHSPC (Optum database of Commercial and Medicare Advantage enrollees) between 2014 and 2019 and 13,324 men with mHSPC (Medicare fee‐for‐service (FFS) enrollees' data) between 2014 and 2017. Findings showed that only 62% of Optum and 52% of Medicare FFS enrollees received any therapy for mHSPC, with ADT alone being utilized in 43% and 46% of Optum and Medicare FFS enrollees, respectively [17]. Freedland et al. also examined the utilization patterns in 1395 men with mHSPC who received at least one treatment modality using data from the Veteran Healthcare Administration (VHA) between 2014 and 2018. The authors found that nearly two‐thirds (63%) of men received ADT only, followed by ADT in combination with ARIs (24%), docetaxel (8%), and abiraterone (5%) [18]. Recently, Heath et al. utilized the IQVIA claims database of 109,607 men with mHSPC identified from 2015 to 2021. Although the authors noted ADT alone being utilized in almost half of the men with mHSPC, there was an increase in the uptake of abiraterone, enzalutamide, or apalutamide over the years 2015 to 2021 [19].

While these studies have highlighted the high prevalence of ADT monotherapy, they focused only on Medicare Advantage enrollees or Medicare fee‐for‐service enrollees using only claims data or relied on VHA data. To fill in the knowledge gap, we evaluated the treatment patterns and factors associated with intensification beyond ADT among older men (aged 67 years and above) with mHSPC in the US using the Surveillance, Epidemiology, and End Results (SEER) Medicare linked data. This study makes a unique contribution by linking two large population‐based databases, namely, cancer surveillance registry (SEER) and FFS Medicare claims. The combined dataset encompassed a diverse range of racial and ethnic groups and provided comprehensive information on patient demographics, cancer diagnostics, and treatment patterns.

## 2 Methods

### 2.1 Study Design

This study has been exempted from the North Texas Regional Institutional Review Board (NTRIRB) for ethical approval. Waiver of informed consent was approved by the NTRIRB for this observational study using secondary data.

This observational retrospective cohort study utilized the SEER‐Medicare linked database over a 6‐year period from 2015 to 2020. The study cohort comprised male Medicare beneficiaries aged 67 years and older at the time of their diagnosis of mHSPC. The diagnosis timeframe spanned from July 2016 to December 2019, selected to coincide with the reporting period of clinical trial findings related to ADT intensification. The index date was defined as the mHSPC diagnosis date, which was extracted from both the registry and claims databases. A look‐back period of 18 months was applied for patients diagnosed with stages I‐III to identify hormone‐sensitive patients and exclude individuals with metastatic castration‐resistant PC (mCRPC). A 12‐month baseline period prior to the index date allowed for the identification of chronic conditions, medication use, polypharmacy, healthcare resource utilization, and associated costs using Medicare claims data. The treatment identification period will span 6 months following the index date, including the diagnosis date, as previous research indicates an average treatment initiation time of approximately 51 days. An additional 4‐month window will be included to capture combination therapies. The follow‐up period will end at the earliest of the last date of insurance disenrollment, patient death, or December 31, 2020, with a minimum expected follow‐up of 12 months post diagnosis, unless the patient dies earlier.

### 2.2 Data Source

Data sources for this study included: (1) SEER cancer data; (2) Medicare Master Beneficiary Summary Files (MBSF); (3) Medicare claims files. The SEER registry data contains clinical, demographic, and cause of death information for cancer cases diagnosed within various locations across the US, representing approximately 34% of the US population. The MBSF files include demographic and enrollment details for each Medicare beneficiary. The Medicare claims files were submitted by various providers and healthcare facilities and contribute detailed billing and service information, including diagnosis and procedure codes, prescription medication information, provider information, service dates, and payment details.

### 2.3 Study Cohort

The study cohort comprised 6850 men diagnosed with mHSPC between July 2016 and December 2019, identified from a larger dataset of 655,074 cancer cases recorded in the SEER Registry from 2008 to 2019 (Appendix 1). We identified mHSPC from two separate sources: (a) men initially diagnosed with metastatic PC (Combined Summary Stage—distant: spread to distant lymph nodes or metastases) in the SEER registry were considered to have de novo mHSPC; (b) to identify recurrent mHSPC cases, a modified version of the published algorithm by Freedland et al. was employed (Appendix 2). This algorithm effectively accommodates scenarios where repeated Prostate‐Specific Antigen (PSA) measurements are unavailable, although it is noted that fewer than 1% of PC patients are identified as having mHSPC based solely on PSA values [20].

Additionally, men must be 67 years and older at mHSPC diagnosis and had uninterrupted FFS Medicare coverage (Parts A, B, and D) for at least 12 months prior to diagnosis and 12 months following diagnosis. The age cutoff was chosen to ensure a sufficient look‐back period, given that Medicare eligibility begins at age 65. The enrollment criteria were crucial for analyzing treatment patterns and healthcare resource utilization. Men with any Health Maintenance Organization (HMO) coverage during these periods were excluded, as their medical claims were not available. Men with multiple cancer diagnoses or who received hospice care prior to mHSPC diagnosis were excluded to ensure that treatment effects can be accurately attributed to mHSPC.

### 2.4 Study Measures

#### 2.4.1 Dependent Variable: mHSPC Initial Treatment

Treatment within 6 months of mHSPC was identified from Medicare claims and classified into three categories: (1) ADT + ARPIs, which included patients who had claims for ARPIs within 30 days prior to or 4 months after the initiation of ADT. Due to limited claims data availability (up to December 2020) following the 2019 approvals of apalutamide and enzalutamide, only descriptive analyses will be conducted for this group. (2) ADT + docetaxel, which consisted of patients with claims for docetaxel within 30 days prior to or 4 months after ADT initiation for de novo mHSPC, or at any time for recurrent mHSPC. (3) ADT monotherapy, which included patients treated exclusively with ADT or in combination with bicalutamide, flutamide, or nilutamide. Patients receiving triplet therapy were excluded from our analysis due to the small number of individuals treated (*N* = 36).

#### 2.4.2 Independent Variables

Baseline characteristics comprised age at diagnosis, race and ethnicity, marital status, region of residence, type of metastasis at diagnosis, number of chronic conditions, and the year of diagnosis. Specifically, age was recorded as a continuous variable reflecting the patient's age at the time of diagnosis, while race and ethnicity were categorized into groups such as White, Black or African American, Hispanic or Latino, Asian, and Other. Marital status was also categorized, encompassing options like Single, Married, Divorced, and Widowed, to capture potential social support differences. Additionally, region was classified into four geographic areas: Northeast, Midwest, South, and West. The analysis distinguishes between de novo metastatic cases, indicating newly diagnosed metastatic disease, and recurrent mHSPC, reflecting a recurrence of previously treated non‐metastatic disease. The total number of chronic conditions reported by patients serves as a continuous variable, providing insight into their overall health status. Lastly, the year of diagnosis was included as a continuous variable to account for potential temporal trends in treatment and outcomes.

In addition to individual characteristics, this study incorporates census‐tract‐level social determinants of health (SDoH) through the Yost Index, a composite measure of socioeconomic status. The Yost Index comprises seven census variables: average educational level, median income, poverty rate, median housing value, median rent, unemployment rate, and employment mix. These variables collectively provide a nuanced understanding of the economic context in which patients reside, highlighting how social determinants may influence health outcomes in mHSPC patients. By integrating these individual and contextual factors, the study aims to identify disparities and better understand the complex interplay of personal and socioeconomic influences on the management of mHSPC.

#### 2.4.3 Other Variable: Next Line of Treatment After Index Therapy

The next line of therapy was defined as the first medication (docetaxel, abiraterone, apalutamide, or enzalutamide) started after the first 6‐month initial treatment identification period, which was not included in the index treatment. Time to next line of therapy was defined as the duration from ADT initiation to the initiation of the next treatment (docetaxel or ARPI) for men who received ADT monotherapy as index treatment; for those who received intensified therapy with docetaxel or ARPI in index therapy, time to next therapy was defined as the time from ADT intensification (initiation of docetaxel or ARPI in 6‐month treatment identification period) to start the next line of therapy. For example, for men who received abiraterone as initial treatment and transitioned to enzalutamide, time to next therapy was defined as the months between abiraterone initiation date and enzalutamide start date.

### 2.5 Statistical Analyses

Descriptive statistics were reported to summarize the demographics, clinical characteristics, and prior treatment histories of the overall study population and the different treatment groups. The Pearson chi‐square test was used to examine factors associated with mHSPC treatment. We applied multinomial logistic regression analysis to estimate the odds ratios (ORs) and 95% confidence intervals (CIs) of receiving ADT intensification with ARPI or chemotherapy versus ADT alone, while controlling for age, gender, baseline health conditions, and SDoH.

## 3 Results

### 3.1 Characteristics of the Study Cohort

Characteristics of the 6850 men in our study cohort were summarized in Table 1. The average age at mHSPC diagnosis was 76.6 years (SD = 7.0). 77.7% of men in the study cohort were non‐Hispanic white (NHW), 8.4% were non‐Hispanic black (NHB), and 6.5% were Hispanic. Additionally, 54.1% were married at PC diagnosis, 17.3% were dual eligible low‐income beneficiaries, 1.7% were in a rural area, and 9.3% were frail as measured by the claim‐based frailty index.

**TABLE 1:** Characteristics of study cohort: stratified by de novo and recurrent mHSPC.

Characteristics	Overall		De novo metastases		Recurrent metastases	
	*N*/mean (SD)	Col %/median (IQR)	*N*/mean (SD)	Col %/median (IQR)	*N*/mean (SD)	Col %/median (IQR)
Cohort size	6850		4042		2808	
Time to treatment from PC, in months						
PC to mHSPC diagnosis	20.95 (36.07)	0.00 (0.00–31.04)	NA	NA	51.32 (40.05)	48.03 (9.04–86.05)
mHSPC to ADT initiation	1.61 (1.18)	1.32 (0.79–2.07)	1.65 (1.11)	1.38 (0.89–2.1)	1.51 (1.32)	1.12 (0.59–1.94)
mHSPC to ADT intensification	2.69 (1.38)	2.43 (1.63–3.62)	2.74 (1.35)	2.47 (1.71–3.65)	2.54 (1.44)	2.24 (1.45–3.39)
ADT initiation to ADT intensification	1.26 (1.36)	0.99 (0.23–1.94)	1.25 (1.32)	0.99 (0.26–1.91)	1.29 (1.49)	1.02 (0.13–2.07)
Age at mHSPC diagnosis	76.6 (7.0)	75 (71–81)	77.43 (7.14)	76 (71–83)	75.4 (6.35)	74 (70–79)
Age at mHSPC diagnosis (categorized)						
67–69	1142	16.7	596	14.7	546	19.4
70–74	1946	28.4	1040	25.7	906	32.3
75–79	1593	23.3	922	22.8	671	23.9
80 or older	2169	31.7	1484	36.7	685	24.4
Race and ethnicity						
NHW	5323	77.7	3128	77.4	2195	78.2
NHB	574	8.4	341	8.4	233	8.3
Hispanic	447	6.5	280	6.9	167	5.9
Other/unknown	506	7.4	293	7.2	213	7.6
Marital status at PC diagnosis^a^						
Married	3706	54.1	2155	53.3	1551	55.2
Widowed/separated/divorced	2191	32	1310	32.4	881	31.4
Never married	953	13.9	577	14.3	376	13.4
Region						
Northeast	2449	35.8	1428	35.3	1021	36.4
South	1761	25.7	1029	25.5	732	26.1
Midwest	482	7	291	7.2	191	6.8
West	2158	31.5	1294	32	864	30.8
Rurality						
Rural	119	1.7	68	1.7	51	1.8
Not rural	6731	98.3	3974	98.3	2757	98.2
Dual eligibility (low‐income) Yes/No						
Dual eligible	1188	17.3	788	19.5	400	14.2
Not dual eligible	5662	82.7	3254	80.5	2408	85.8
YOST index quantiles						
Missing	198	2.9	108	2.7	90	3.2
Quintile 1	789	11.5	491	12.1	298	10.6
Quintile 2	953	13.9	585	14.5	368	13.1
Quintile 3	1144	16.7	699	17.3	445	15.8
Quintile 4	1503	21.9	893	22.1	610	21.7
Quintile 5	2263	33	1266	31.3	997	35.5
PSA at PC diagnosis						
Missing	3385	49.4	1818	45	1567	55.8
< 4	199	2.9	87	2.2	112	4
4–10	996	14.5	318	7.9	678	24.1
11–20	580	8.5	308	7.6	272	9.7
> 20	1690	24.7	1511	37.4	179	6.4
PSA Level based on non‐missing observations	41.1 (39.2)	19 (8–98)	56.27 (39.61)	55.95 (14.3–98)	13.82 (17.79)	7.9 (5.6–13.5)
Gleason score at PC diagnosis						
Missing	3853	56.2	2398	59.3	1455	51.8
Glsn LE7	1114	16.3	303	7.5	811	28.9
Glsn GE8	1883	27.5	1341	33.2	542	19.3
Gleason clinical score based on non‐missing observations	8 (1.2)	8 (7–9)	8.48 (0.99)	9 (8–9)	7.44 (1.07)	7 (7–8)
Risk group based on Gleason score and PSA values^c^						
Missing	3161	46.1	1723	42.6	1438	51.2
Not high risk	1458	21.3	531	13.1	927	33
High risk	2231	32.6	1788	44.2	443	15.8
Claim‐based spread of Metastasis^b^						
Bone only	2969	43.3	1727	42.7	1242	44.2
Bone & visceral	195	2.8	134	3.3	61	2.2
Visceral only	298	4.4	94	2.3	204	7.3
Lymph node only	1052	15.4	336	8.3	716	25.5
Other	2336	34.1	1751	43.3	585	20.8
Claim‐based frailty index (CFI)	0.2 (0.06)	0.15 (0.12–0.19)	0.16 (0.06)	0.15 (0.11–0.19)	0.16 (0.06)	0.15 (0.12–0.19)
CFI < 0.25	6211	90.7	3675	90.9	2536	90.3
CFI ≥ 0.25	639	9.3	367	9.1	272	9.7
Chronic conditions						
Hypertension	5260	76.8	3064	75.8	2196	78.2
High cholesterol	4884	71.3	2763	68.4	2121	75.5
Arthritis	3340	48.8	1922	47.6	1418	50.5
Heart disease	2499	36.5	1483	36.7	1016	36.2
Coronary artery disease	2459	35.9	1441	35.7	1018	36.3
Diabetes	2315	33.8	1319	32.6	996	35.5
Osteoarthritis	2013	29.4	1146	28.4	867	30.9
Cardiac arrhythmia	1855	27.1	1107	27.4	748	26.6
Chronic obstructive pulmonary disease	1390	20.3	792	19.6	598	21.3
Thyroid	1249	18.2	710	17.6	539	19.2
Chronic kidney disease	1249	18.2	786	19.4	463	16.5
Congestive heart failure	1101	16.1	701	17.3	400	14.2
Anxiety	675	9.9	382	9.5	293	10.4
Depression	671	9.8	397	9.8	274	9.8
Gout	602	8.8	362	9	240	8.5
Asthma	473	6.9	260	6.4	213	7.6
Stroke	323	4.7	192	4.8	131	4.7
Osteoporosis	274	4	138	3.4	136	4.8
HIV	199	2.9	114	2.8	85	3
Rheumatoid arthritis	169	2.5	93	2.3	76	2.7
Hepatitis	167	2.4	100	2.5	67	2.4
Multimorbidity, by number of conditions						
Hi Multimorbidity (7 or more)	1567	22.9	933	23.1	634	22.6
Moderate Multimorbidity (3–6)	3646	53.2	2070	51.2	1576	56.1
No Multimorbidity (0–2)	1637	23.9	1039	25.7	598	21.3
Bone health agents—baseline						
Any bone health agents	130	1.9	69	1.7	61	2.2
Denosumab	46	0.7	21	0.5	25	0.9
Alendronate	60	0.9	34	0.8	26	0.9
Baseline healthcare resource use						
IP use	1289	18.8	758	18.8	531	18.9
ER use	2567	37.5	1605	39.7	962	34.3
Diagnostic radiologist visit	5044	73.6	2772	68.6	2272	80.9
Urologist visit	4439	64.8	2384	59	2055	73.2
Oncologist visit	1492	21.8	363	9	1129	40.2
Other specialist visit	4917	71.8	2804	69.4	2113	75.2
PCP visit	6138	89.6	3562	88.1	2576	91.7
Pain specialist visit	1897	27.7	988	24.4	909	32.4
Psychologist visit	327	4.8	194	4.8	133	4.7
Rheumatologist visit	225	3.3	117	2.9	108	3.8
Ortho surgeon visit	1240	18.1	701	17.3	539	19.2
Gastroenterologist visit	1246	18.2	624	15.4	622	22.2
Anesthesiologist visit	1828	26.7	947	23.4	881	31.4
Polypharmacy	3140	45.8	1928	47.7	1212	43.2

^a^ Missing values in marital status were randomly imputed.

^b^ Other: metastasis recorded in SEER without a corresponding claim indicating a specific site or had claim‐based metastasis sites that did not fall into the predefined categories of bone, visceral, or lymph node metastases.

^c^ High risk was defined as Gleason score ≥ 8, or PSA > 20 at PC diagnosis.

The majority (*N* = 4042, 59.0%) had de novo metastases; among those who had recurrent metastases, the median time from PC diagnosis to metastasis was 48.0 months (IQR 9.0–86.1). Among the study cohort, 43.3% had bone metastasis only, 2.8% and 4.4% had visceral metastasis with and without bone metastasis, and 15.4% had lymph node metastasis only.

### 3.2 mHSPC Treatment Pattern

**FIGURE 1:**
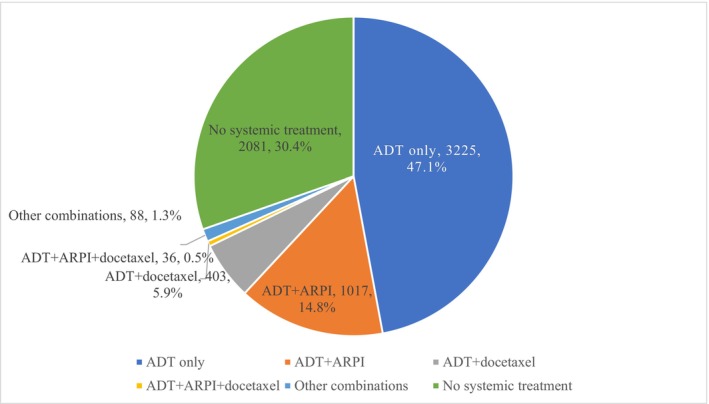
Percent treated with ADT only, ADT + ARPI, and ADT + docetaxel among men aged 67 or older, and diagnosed with mHSPC between July 2016 and December 2019 (Total *N* = 6850).

Among 6850 men in our study cohort, 2081 (30.4%) received no systemic treatment within 6 months of mHSPC diagnosis; 3225 (47.1%) received ADT monotherapy; 1017 (14.8%) received ADT plus ARPI, and 403 (5.9%) received ADT plus docetaxel; < 2% received triplet therapy or other treatment combinations (Figure 1).

**FIGURE 2:**
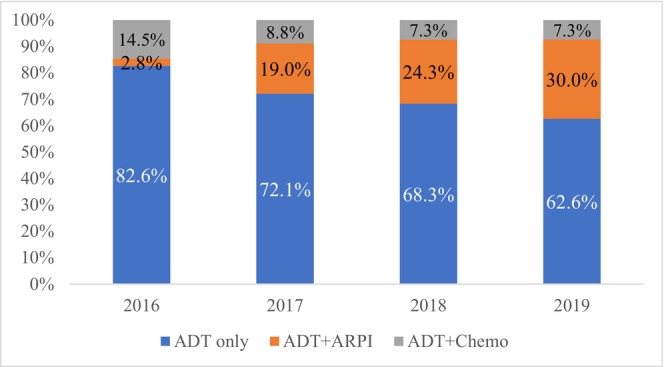
Trends in mHSPC treatment pattern in men aged 67 or older, diagnosed with mHSPC in 2016–2019, and treated with ADT (total *N* = 4645). 2016 year includes only patients who started treatment on or after July 1, 2016. Triplet therapy was excluded due to the small number of individuals receiving treatment. ADT, androgen deprivation therapy; ARPI, androgen receptor pathway inhibitor; mHSPC, metastatic hormone‐sensitive prostate cancer.

Among 4645 men treated with ADT, the use of ADT + ARPI increased from 19.0% to 30.0% from 2017 to 2019, while treatments with ADT alone decreased from 72.1% to 62.6% and ADT plus docetaxel decreased from 8.8% to 7.3% (Figure 2). Notably, disparities were observed based on race and socioeconomic factors. Among non‐Hispanic whites, 22.5% received ADT plus ARPI compared to 15.5% among non‐Hispanic blacks (Table 2). Furthermore, individuals in the highest Yost Index quintile had higher rates of ADT plus ARPI (26.0%) and ADT plus docetaxel (9.2%) than those in the lowest quintile (15.1% and 6.3%, respectively).

**TABLE 2:** Baseline characteristics of individuals with mHSPC and treated with ADT by treatment.

Characteristics	ADT		ADT+ Docetaxel		ADT + ARPI		*p*
	*N*/mean (SD)	Row %/median (IQR)	*N*/mean (SD)	Row %/median (IQR)	*N*/mean (SD)	Row %/median (IQR)	
Cohort size	3225	69.4	403	8.7	1017	21.9	
PC to mHSPC diagnosis	15.78 (31.75)	0 (0–5.95)	10.16 (27.1)	0 (0–0)	14.5 (31.26)	0 (0–2.01)	
mHSPC to ADT initiation	1.69 (1.24)	1.35 (0.82–2.24)	1.42 (0.95)	1.22 (0.79–1.74)	1.44 (1.01)	1.22 (0.76–1.87)	
ADT use to ADT Intensification	NA	NA	1.31 (1.37)	1.08 (0.43–2.01)	1.23 (1.36)	0.95 (0.2–1.91)	
Age at mHSPC diagnosis	77.7 (7.09)	77 (72–83)	72.93 (4.65)	72 (69–76)	76.35 (6.13)	76 (71–81)	
Race and ethnicity							0.011
NHW	2527	68.6	325	8.8	830	22.5	
NHB	257	78.1	21	6.4	51	15.5	
Hispanic	219	73.2	23	7.7	57	19.1	
Other/unknown	222	66.3	34	10.1	79	23.6	
Marital status at PC diagnosis							< 0.001
Married	1700	67.0	243	9.6	593	23.4	
Widowed/divorced/separated	1085	72.9	119	8.0	285	19.1	
Never married	440	71.0	41	6.6	139	22.4	
Region							< 0.001
Northeast	1115	68.8	122	7.5	384	23.7	
South	842	71.1	116	9.8	226	19.1	
Midwest	255	71.8	43	12.1	57	16.1	
West	1013	68.2	122	8.2	350	23.6	
Dual eligibility							< 0.001
Medicaid/medicare	552	75.2	38	5.2	144	19.6	
Medicare only	2673	68.3	365	9.3	873	22.3	
YOST quintile							< 0.001
Quintile 1	396	78.6	32	6.3	76	15.1	
Quintile 2	473	71.9	66	10.0	119	18.1	
Quintile 3	571	71.3	65	8.1	165	20.6	
Quintile 4	720	69.4	85	8.2	232	22.4	
Quintile 5	986	64.8	140	9.2	395	26.0	
PSA level at diagnosis	43.6 (38.92)	23.7 (8.8–98)	57.59 (40.51)	66.8 (12.7–98)	52.61 (39.95)	45.25 (11–98)	
Gleason score at diagnosis	8.16 (1.06)	8 (7–9)	8.59 (0.94)	9 (8–9)	8.45 (1.04)	9 (8–9)	
Risk group based on Gleason score and PSA values^c^							< 0.001
High risk	617	75.9	44	5.4	152	18.7	
Not high risk	1185	65.4	197	10.9	430	23.7	
Multimorbidity, by number of conditions							< 0.001
Hi multimorbidity (7 or more)	782	76.2	45	4.4	199	19.4	
Moderate multimorbidity (3–6)	1728	69.4	226	9.1	536	21.5	
No multimorbidity (0–2)	715	63.3	132	11.7	282	25.0	
Baseline claim‐based frailty index (CFI)	0.16 (0.06)	0.15 (0.12–0.19)	0.14 (0.04)	0.13 (0.11–0.16)	0.15 (0.05)	0.14 (0.11–0.17)	
Baseline Charlson comorbidity index	2.05 (2.11)	1 (0–3)	1.26 (1.48)	1 (0–2)	1.73 (1.94)	1 (0–3)	
Baseline care fragmentation index	0.74 (0.16)	0.79 (0.70–0.85)	0.71 (0.19)	0.76 (0.64–0.84)	0.74 (0.17)	0.79 (0.68–0.85)	
Claim‐based spread of metastasis							< 0.001
Bone only	1497	66.9	204	9.1	536	24.0	
Bone and visceral	83	57.6	24	16.7	37	25.7	
Visceral only	72	63.2	17	14.9	25	21.9	
Lymph node only	521	80.4	21	3.2	106	16.4	
N/A	1052	70.0	137	9.1	313	20.8	
Healthcare resource use							
IP use^a^	619	74.8	45	5.4	164	19.8	< 0.001
No IP use	2606	68.3	358	9.4	853	22.3	
ER use^a^	1268	73.0	120	6.9	348	20.0	< 0.001
No ER use	1957	67.3	283	9.7	669	23.0	
Diagnostic radiologist visit^b^	2507	66.7	368	9.8	881	23.5	< 0.001
No diagnostic radiologist Visit	718	80.8	35	3.9	136	15.3	
Urologist visit^b^	2455	69.7	305	8.7	760	21.6	0.663
No urologist visit	770	68.4	98	8.7	257	22.8	
Oncologist visit^b^	2206	64.1	367	10.7	868	25.2	< 0.001
No oncologist Visit	1019	84.6	36	3.0	149	12.4	
Other specialist visit^b^	1438	68.5	189	9.0	471	22.4	0.482
No other specialist visit	1787	70.2	214	8.4	546	21.4	
Healthcare resource use							
PCP visit^b^	2200	68.3	289	9.0	730	22.7	0.054
No PCP visit	1025	71.9	114	8.0	287	20.1	
Pain specialist visit^b^	516	64.2	95	11.8	193	24.0	< 0.001
No pain specialist visit	2709	70.5	308	8.0	824	21.5	
Ortho surgeon visit^b^	202	63.7	32	10.1	83	26.2	0.073
No ortho surgeon visit	3023	69.8	371	8.6	934	21.6	
Gastroenterologist visit^b^	223	62.6	40	11.2	93	26.1	0.013
No gastroenterologist visit	3002	70.0	363	8.5	924	21.5	
Anesthesiologist visit^b^	491	64.0	92	12.0	184	24.0	< 0.001
No anesthesiologist visit	2734	70.5	311	8.0	833	21.5	
Polypharmacy (≥ 6 drugs)^a^	1538	71.2	158	7.3	464	21.5	0.005
No polypharmacy	1687	67.9	245	9.9	553	22.3	

Abbreviations: ADT, androgen deprivation therapy; ARPI, androgen receptor pathway inhibitor; BHA, bone health agent; CCI, Charlson Comorbidity Index; CHF, congestive heart failure; CVD, cerebrovascular disease; IQR, interquartile range; MI, myocardial infarction; MMC, Medicaid Managed Care; PVD, peripheral vascular disease; SD, standard deviation.

^a^ Baseline IP use, ER use, Polypharmacy 12 months before mHSPC diagnosis.

^b^ Any visit within 30 days before and 30 days after ADT initiation. YOST index has missing data and will not add to total numbers (not presented).

^c^ High risk was defined as Gleason score ≥ 8, or PSA > 20 at PC diagnosis.

Additionally, individuals with higher PC risk, indicated by PSA levels and Gleason scores, were less likely to receive ADT plus ARPI (18.7%) and ADT plus docetaxel (5.4%) compared to not high‐risk individuals (23.7% and 10.9%, respectively). Those with bone and visceral metastases were more likely to receive ADT plus ARPI (25.7%) and ADT plus docetaxel (16.7%). Moreover, individuals with high multimorbidity exhibited lower rates of receiving ADT plus ARPI (19.4%) and ADT plus docetaxel (4.4%) compared to those without multimorbidity (25.0% and 11.7%). The mean Charlson comorbidity score was found to be lower among those receiving intensified ADT compared to those on ADT alone. Physician specialty of visits near the mHSPC diagnosis date (within 30 days before and 30 days after index date) was also associated with treatment category. Men who had oncologist visits had higher rates of receiving ADT + ARPI (25.2% vs. 12.4%) or ADT + docetaxel (10.7% vs. 3.0%) compared with those who had no oncologist visit; however, no difference was observed between men with and without urologist visits near mHSPC diagnosis.

### 3.3 Baseline Characteristics Associated With ADT Combined With ARPI and/or Docetaxel

In multinomial logistic regression analysis (Table 3), patients diagnosed with de novo mHSPC exhibited a greater propensity to receive ADT in conjunction with docetaxel (adjusted odds ratio [aOR] = 2.73, 95% confidence interval [CI] = [2.07, 3.60]) or ADT combined with ARPI (aOR = 1.56, 95% CI = [1.32, 1.84]) compared to those receiving ADT alone. Conversely, non‐Hispanic Black men demonstrated a decreased likelihood of receiving ADT combined with ARPI (aOR = 0.71, 95% CI = [0.51, 0.98]). Moreover, dual eligible low‐income patients exhibited a reduced likelihood of receiving ADT combined with docetaxel (aOR = 0.59, 95% CI = [0.40, 0.87]). Socioeconomic status emerged as a significant factor, with individuals residing in the lowest socioeconomic quintile (YOST Quintile 1) demonstrating a diminished likelihood of receiving either ADT combined with docetaxel (aOR = 0.62, 95% CI = [0.39, 0.97]) or ADT combined with ARPI (aOR = 0.54, 95% CI = [0.40, 0.74]) relative to those in the highest quintile (YOST Quintile 5). Furthermore, higher frailty was correlated with a lower likelihood of receiving intensified therapies; specifically, one percentage point increase in frailty index is associated with a 7% and 3% decrease in the odds of receiving ADT combined with docetaxel (aOR = 0.93, 95% CI = [0.91, 0.95]) and ADT combined with ARPI (aOR = 0.97, 95% CI = [0.96, 0.98]), respectively. Age at diagnosis also influenced treatment modalities; patients aged 75 years and older were less likely to receive ADT combined with docetaxel (aOR = 0.41, 95% CI = [0.32, 0.52]), while the likelihood of receiving ADT combined with ARPI did not show a statistically significant difference (aOR = 0.97, 95% CI = [0.78, 1.20]).

**TABLE 3:** Adjusted odds ratios and 95% confidence intervals from multinomial logistic regressions: ADT + docetaxel, ADT + ARPI (reference group: ADT only).

Characteristics	ADT + Docetaxel			ADT + ARPI		
	aOR	95% CI	*p*	aOR	95% CI	*p*
mHSPC diagnosis year (continuous variable, 2016 is Year 0)	0.87	[0.79, 0.97]	0.0100	1.64	[1.52, 1.78]	< 0.001
De novo metastases (reference group: recurrent)	2.73	[2.07, 3.60]	< 0.001	1.56	[1.32, 1.84]	< 0.001
Age ≥ 75 years at mHSPC diagnosis (reference group: age 67–74 years)	0.41	[0.32, 0.52]	< 0.001	0.97	[0.78, 1.20]	0.7700
Race and ethnicity (reference group: NHW)						
NHB	0.68	[0.42, 1.11]	0.1229	0.71	[0.51, 0.98]	0.0369
Hispanic	1.04	[0.64, 1.68]	0.8771	0.89	[0.64, 1.23]	0.4780
Other/unknown	1.16	[0.78, 1.74]	0.4674	0.99	[0.75, 1.32]	0.9524
Marital status at PC diagnosis (reference group: married)						
Widowed/divorced/separated	0.87	[0.68, 1.11]	0.2514	0.76	[0.64, 0.90]	0.0012
Never married	0.77	[0.54, 1.10]	0.1512	0.92	[0.73, 1.14]	0.4394
Region at PC diagnosis (reference group: northeast)						
South	1.37	[1.02, 1.85]	0.0362	0.90	[0.73, 1.11]	0.3181
Midwest	1.49	[0.99, 2.22]	0.0547	0.65	[0.47, 0.90]	0.0100
West	1.03	[0.78, 1.37]	0.8111	0.98	[0.82, 1.17]	0.7931
Urban (reference group: rural)	1.35	[0.56, 3.25]	0.4988	1.22	[0.64, 2.33]	0.5444
YOST index quintile^a^ (reference group: Quintile 5)						
Quintile 1	0.62	[0.39, 0.97]	0.0364	0.54	[0.40, 0.74]	< 0.001
Quintile 2	0.99	[0.71, 1.40]	0.9652	0.68	[0.53, 0.88]	0.0031
Quintile 3	0.78	[0.56, 1.09]	0.1434	0.77	[0.61, 0.96]	0.0195
Quintile 4	0.82	[0.61, 1.10]	0.1882	0.83	[0.68, 1.01]	0.0611
Dual eligibility in baseline period (medicare/medicaid) (reference group: medicare only)	0.59	[0.40, 0.87]	0.0072	1.07	[0.85, 1.35]	0.5723
Frailty index in baseline period^b^	0.93	[0.91, 0.95]	< 0.001	0.97	[0.96, 0.98]	< 0.001

^a^ Missing indicator for YOST index included but not presented in the table.

^b^ Frailty index is expressed as a percentage.

### 3.4 Next Line of Treatment After Index Therapy

**FIGURE 3:**
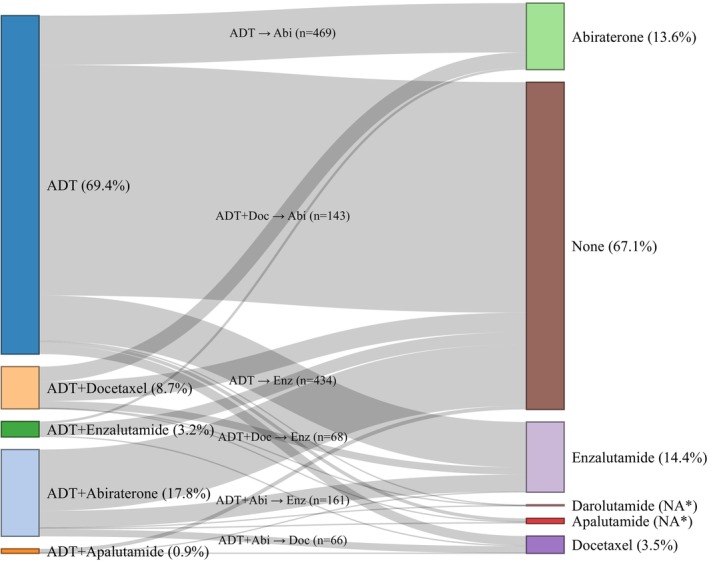
Flow of subsequent treatments after index therapy in patients treated with ADT among men aged 67 or older, diagnosed with mHSPC in 2016–2019, and treated with ADT. *Percentages were suppressed due to small cell sizes.

Among the 4645 individuals treated with ADT, 1526 (32.9%) initiated another systemic therapy during the follow‐up period (Figure 3; Appendix 3). The most common subsequent treatment following the index therapy was ARPIs. Among the 3225 men initially treated with ADT monotherapy, 31.8% (*N* = 1028) transitioned to another therapy, with abiraterone (*N* = 469) and enzalutamide (*N* = 434) being the most frequent next treatments (Figure 3; Appendix 3). By comparison, 27.6% (*N* = 281) of men who began treatment with ADT combined with an ARPI initiated another therapy, most commonly transitioning from abiraterone to enzalutamide (*N* = 161) or from abiraterone to docetaxel (*N* = 66). For those initially treated with ADT combined with chemotherapy, more than half (53.8%, *N* = 217) transitioned to another therapy, with abiraterone (*N* = 143) and enzalutamide (*N* = 68) being the most common choices (Figure 3).

The average time from ADT monotherapy initiation to the next treatment was 14.9 months for abiraterone and 15.5 months for enzalutamide (Appendix 3). For men who received initial treatment with docetaxel, the average time to subsequent therapy was 12.9 months for abiraterone and 16.9 months for enzalutamide. Among men initially treated with an ARPI, the average durations to abiraterone and enzalutamide were 11.2 and 11.5 months, respectively.

## 4 Discussion

In this study, we analyzed initial treatment patterns among older men diagnosed with mHSPC in the US between 2016 and 2020. The nationwide cancer SEER registry data linked with Medicare claims data enabled us to examine a large number of cancer cases with detailed treatment information, ensuring high accuracy in both the identification of mHSPC cases and the tracking of treatment regimens. The linked dataset incorporates a comprehensive set of demographics, clinical, and SDoH factors among a diverse group of individuals, allowing for an in‐depth exploration of treatment patterns and disparities.

ADT monotherapy was still prevalent, accounting for 67.6% of systemic mHSPC treatments over the study period. Single‐modality treatment among older adults may reflect clinical considerations, such as contraindications to intensified therapy, concerns about adverse effects, limited survival benefits in patients with poor functional status or multiple comorbidities, and cost‐related barriers [21]. Among men who received systemic treatment, intensification with docetaxel decreased from 14.5% to 8.8% during 2016–2017, and remained stable at 7.3% during 2018–2020. Conversely, ARPI treatment surged from 2.8% to 30.0% over the study period. Our findings align with other studies using Veterans Health Administration data [18], chart reviews [22], open claims [19], and clinical electronic health records [23]. These changes in treatment patterns were likely driven by approval and guideline updates recommending intensification and incorporating novel ARPIs. Despite a significant increase in the rate of intensified therapy, the high prevalence of ADT monotherapy underscores gaps in receiving approved and guideline‐recommended treatment.

Aligning with existing studies [24], we found that men who were younger, had de novo mHSPC, higher PSA and Gleason scores at PC diagnosis, and had visceral metastases were more likely to receive treatment intensification. Additionally, treatment patterns varied by physician specialty, with oncologists, who often manage older, higher comorbidity patients, being more likely to prescribe combination therapies than urologists.

Our study expands previous literature by highlighting disparities in mHSPC treatment based on race and ethnicity, marital status, region, income, and neighborhood social‐economic status as captured by the YOST index. Compared with Non‐Hispanic White men, Non‐Hispanic black men had lower odds of receiving intensified therapy with ARPI. In contrast, such disparities did not exist in other ethnic groups. Our findings in racial disparity align with recent studies [18, 23, 25]. Additionally, men residing in the Midwest and those who were widowed, divorced, or separated were less likely to receive ARPI, and dual eligible low‐income individuals had reduced odds of receiving treatment intensification with docetaxel. A novel finding from our study was the gradient decrease in the odds of receiving ADT + ARPI from the top YOST Index Quintiles to the bottom, indicating neighborhood socioeconomic status remained an independent predictor of receiving novel therapy after adjusting for demographic, clinical, and other SDoH characteristics.

The observed disparities in mHSPC treatment may stem from systemic, neighborhood, and patient‐level factors. NHB, unmarried, low‐income individuals may face barriers such as high out‐of‐pocket costs for intensified therapies [26], restrictive formulary policies or delayed approval for treatment intensification [27], limited health literacy [28], and lack of caregiving support [29]. Furthermore, men living in neighborhoods with lower socioeconomic status may experience limited access to oncology specialists or comprehensive cancer centers [30]. These inequities are particularly concerning given that disadvantaged groups consistently experience poorer outcomes, such as earlier disease progression and worse OS [31, 32].

Reducing disparities in treatment access is critical to narrowing survival gaps and improving health equity among older men with mHSPC. Ensuring equitable access to guideline‐recommended therapies requires a multifaceted approach, including targeted patient outreach, policies aimed at improving care coordination, and programs designed to address patients' social needs. The Inflation Reduction Act offers a promising step toward mitigating financial toxicity associated with advanced cancer treatment by capping total out‐of‐pocket costs at $2000 [33]. Such measures are essential for advancing equity and improving outcomes for older adults with advanced PC.

A noteworthy finding is that nearly one‐third of patients initially treated with ADT (whether as monotherapy or in combination) eventually received additional systemic treatment, with ARPIs emerging as the most common next line option. The average time from ADT to subsequent therapy varied by initial treatment category. Patients who were on ADT only experienced a longer duration before transition to the next line of therapy, suggesting a need to intensify therapy earlier and facilitate access to other lines of treatment. Variations in duration may also reflect differences in disease severity, response profile, or patient selection. These findings suggest the need for personalized treatment strategies based on individual risk profiles and the initial choice of therapy [34].

## 5 Limitations

This study has several limitations that should be considered when interpreting the findings. First, due to a 3‐year lag in data release from NCI and Medicare, our data may not fully capture the adoption and use of therapies that became widely available only from late 2019 onward. For example, Darulutamide in combination with docetaxel was approved for mHSPC treatment in 2022, and triplet therapy was recommended in the 2023 ASCO treatment guidelines [35]. Second, our study cohort consisted of older Medicare beneficiaries only. As our analysis focused on older men within the Medicare population, the results may not be directly generalizable to younger adults with early onset PC, who may exhibit distinct PC phenotypes [36, 37]. Third, missingness in key clinical variables, such as baseline PSA level and Gleason score, may not be random and could introduce exclusion bias. Finally, the claims‐based algorithm used to identify recurrent mHSPC prioritizes specificity over sensitivity and therefore may miss some mHSPC patients who progressed from a non‐metastatic stage [20]. In contrast, all patients initially diagnosed with metastases will be identified as de novo mHSPC, which may introduce some bias toward positive identification. The absence of repeated measurement of PSA levels may introduce some imprecision in identifying mHSPC. However, as noted by Freedland and colleagues, the imprecision will be insignificant [20]. Additionally, claim‐based sites of metastasis demonstrated limited accuracy, as reported in an old study [35]; however, later studies comparing electronic health records to claims data have reported improved concordance [38].

## 6 Conclusion

Overall, 3 in 10 older men with mHSPC received no systemic drug treatment in 2016–2020. Although there was a gradual uptake of ARPIs, monotherapy with ADT was still highly prevalent, suggesting the integration of intensified treatment for mHSPC is still suboptimal despite insurance coverage and guideline recommendations. There is a need for targeted interventions to increase the utilization of guideline‐recommended treatments for mHSPC to improve clinical outcomes and reduce racial and socio‐economic disparities in the management of mHSPC.

## Author Contributions

**Bo Zhou:** conceptualization, methodology, data curation, writing – original draft, writing – review and editing, formal analysis, visualization, investigation, validation, software. **Amit D. Raval:** funding acquisition, writing – review and editing, methodology, project administration, investigation, conceptualization, validation. **Yifan Zhang:** formal analysis, writing – review and editing, visualization, validation, investigation, software. **Matthew J. Korn:** writing – review and editing, methodology, validation. **Niculae Constantinovici:** writing – review and editing, validation. **Nethra Sambamoorthi:** formal analysis, writing – review and editing, software. **Rafia Rasu:** investigation, writing – review and editing, validation, methodology. **Natasha Littleton:** investigation, writing – review and editing, validation. **Usha Sambamoorthi:** conceptualization, funding acquisition, methodology, data curation, supervision, writing – review and editing, writing – original draft, project administration, resources, software, formal analysis, validation, visualization, investigation.

## Conflicts of Interest

Author financial disclosure: Amitkumar Raval, Matthew J. Korn, Natasha Littleton, and Niculae Constantinovici are employees and stockholders of Bayer. Bo Zhou, Nethra Sambamoorthi, Rafia Rasu, and Usha Sambamoorthi received research funding from Bayer to conduct the study.

## Data Availability

The data that support the findings of this study are available from SEER Medicare. Restrictions apply to the availability of these data, which were used under license for this study. Data are available from https://healthcaredelivery.cancer.gov/seermedicare/ with the permission of SEER Medicare.

## Appendix 1 Cohort Selection

**Table:**

Steps	Cohort selection criteria	Sample size
1	Incident Primary Prostate Cancer (PC) from SEER Registry Data 2008–2019	655,074
2	Metastatic PC (mPC) identified from SEER Registry or Medicare Claims Jul 2016‐ Dec 2019	36,414
3	Alive 6 months after initial mPC diagnosis and eligible to receive curative cancer care	30,078
4	Castration naïve or ICD‐code for hormone sensitivity (Z19.1)	25,047
5	Confirmed hormone sensitivity from Medicare claims, and received no treatment for metastatic castration‐resistant PC (mCRPC)	24,575
6	Age 67 or above at metastatic Hormone Sensitive PC (mHSPC) diagnosis	19,671
7	Continuous enrollment in fee‐for‐service (FFS) Parts A/B/D 12 months prior to and 6 months after mHSPC diagnosis	6850
7a	Received systemic treatment within 6 months after mHSPC diagnosis	4769
7b	Treated with ADT	4681
7c	Treated with ADT but without triplet therapy	4645

Abbreviations: ADT, androgen deprivation therapy; ICD‐10‐CM, International Classification of Diseases 10th revision, clinical modification; mCRPC, metastatic castration‐resistant prostate cancer; mHSPC, metastatic hormone‐sensitive prostate cancer; mPC, metastatic prostate cancer.

## Appendix 2 Algorithm to Identify Men With mHSPC

Recurrent mHSPC will be identified based on the first metastasis Medicare claim for men initially registered with non‐metastatic PC (AJCC stage I–III or M0 stage) in the SEER registry, using the published algorithm by Freedland et al. [34]:

Step 1: Establish Index Date.

- Identify metastatic disease (ICD‐9: 196.xx‐199.1x, 209.7x, ICD10‐ C77. xx.‐C80.0x, C7B) to establish index date.
- The index date is the first observed date associated with metastatic disease after PC diagnosis.

Step 2: Identify Hormone sensitivity (Satisfy any one of the criteria).

- Criterion #1: Identify hormone sensitivity (≥ 1 claim with ICD10 (ICD‐10‐CM Z19.1)) in 12‐month window prior to index date.
- Criterion #2: Hormone/castration naive, defined as no claim for surgical or medical castration within 18 months before the index date.

Step 3: Confirm Hormone sensitivity: Exclude those with any of the following criteria.

- Criterion #1: Diagnosis code (ICD‐10: CM Z19.2) indicating hormone resistance during the mHSPC identification period.
- Criterion #2: Rising PSA levels (ICD‐10 CM R97.21) following surgical castration during the mHSPC identification period.
- Criterion #3: Rising PSA levels (ICD‐10 CM R97.21) following medical castration during the mHSPC identification period. Medical castration is defined as ≥ 90 days of continuous ADT use.
- Criterion #4: Had surgical castration at least 90 days prior to the index date.
- Criterion #5: Had continuous ADT throughout 90 days (with no gap > 30 days) prior to the index date. HCPCS J codes, dosage designation (1, 3, 4) from the NCH files or revenue center unit count from OUTSAF files will be used to calculate hormone therapy for men with prostate cancer as per guidance from SEER (https://healthcaredelivery.cancer.gov/seermedicare/considerations/prostate.html).
- Criterion #6: ≥ 1 claim of the following medications use—Pembrolizumab, Sipuleucel‐T, Radium‐223, Lutetium Lu 177 vipivotide tetraxetan, Rucaparib, Olaparib, abiraterone before 02/2018, enzalutamide before 12/2019) prior to the index date.

## Appendix 3 Number of Months to Next Non‐Index Therapy

**Table:**

Older adults (age ≥ 67 years) with mHSPC						
SEER‐medicare linked database (2015–2020)						
	Overall cohort	ADT only	ADT + Docetaxel	ADT + ARPI	ADT+ ABI	ADT+ ENZA
Cohort size	4645	3225	403	1017	827	148
Number of individuals received non‐index therapy	1526	1028	217	281	242	32
Time to next treatment in months						
ARPIs	13.36	14.03	12.47	11.25	11.40	11.24
Abiraterone	14.38	14.92	12.85	11.24	NA	11.24
Apalutamide	17.58	19.08	##	11.92	11.92	##
Darolutamide	24.07	27.32	##	##	##	##
Enzalutamide	14.87	15.54	16.90	11.52	11.68	NA
Treatment patterns among men with mCRPC						
Number of individuals who progressed to mCRPC	1358	886	169	303	249	44
Number of individuals received non‐index therapy	783	507	117	159	139	18
Time to next treatment in months						
ARPI	14.04	14.65	12.70	12.83	12.95	##
Abiraterone	15.40	16.07	13.45	##	NA	##
Apalutamide	20.28	20.61	##	##	##	##
Darolutamide	27.01	##	##	##	##	##
Enzalutamide	16.00	16.42	18.44	13.01	12.78	NA

*Note:* ## indicates cell sizes < 11 and suppressed as per CMS policy.

Abbreviation: NA, not applicable.
